# Efficient ATP synthesis by thermophilic *Bacillus* F_o_F_1_-ATP synthase

**DOI:** 10.1111/j.1742-4658.2011.08191.x

**Published:** 2011-08

**Authors:** Naoki Soga, Kazuhiko Kinosita, Masasuke Yoshida, Toshiharu Suzuki

**Affiliations:** 1 Department of Physics, Faculty of Science and Engineering, Waseda UniversityTokyo, Japan; 2 ATP Synthesis Regulation Project, ICORP, Japan Science and Technology Agency (JST)Tokyo, Japan; 3 Department of Molecular Bioscience, Kyoto Sangyo UniversityKyoto City, Japan

**Keywords:** ATP synthesis, Michaelis–Menten constants, reconstitution, temperature, TF_o_F_1_

## Abstract

F_o_F_1_-ATP synthase (F_o_F_1_) synthesizes ATP in the F_1_ portion when protons flow through F_o_ to rotate the shaft common to F_1_ and F_o_. Rotary synthesis in isolated F_1_ alone has been shown by applying external torque to F_1_ of thermophilic origin. Proton-driven ATP synthesis by thermophilic *Bacillus* PS3 F_o_F_1_ (TF_o_F_1_), however, has so far been poor *in vitro*, of the order of 1 s^−1^ or less, hampering reliable characterization. Here, by using a mutant TF_o_F_1_ lacking an inhibitory segment of the ε-subunit, we have developed highly reproducible, simple procedures for the preparation of active proteoliposomes and for kinetic analysis of ATP synthesis, which was driven by acid–base transition and K^+^-diffusion potential. The synthesis activity reached ∼ 16 s^−1^ at 30 °C with a *Q*_10_ temperature coefficient of 3–4 between 10 and 30 °C, suggesting a high level of activity at the physiological temperature of ∼ 60 °C. The Michaelis–Menten constants for the substrates ADP and inorganic phosphate were 13 μm and 0.55 mm, respectively, which are an order of magnitude lower than previous estimates and are suited to efficient ATP synthesis.

## Introduction

F_o_F_1_-ATP synthase (F_o_F_1_) synthesizes the majority of cellular ATP from ADP and P_i_ in respiratory and photosynthetic organisms [1–4]. It consists of two portions, membrane-embedded F_o_ and soluble F_1_, and, when isolated, F_o_ works as a proton (Na^+^ in some bacteria) conductor and F_1_ as an ATPase (F_1_-ATPase). In the simplest version of bacterial F_o_F_1_, the subunit compositions are ab_2_c_10–15_ (F_o_) and α_3_β_3_γδε (F_1_). Both F_o_ and F_1_ are rotary motors, F_o_ being driven by proton flow and F_1_ by ATP hydrolysis. An oligomer ring of c-subunits (c-ring) and γε subunits are considered to rotate together, forming a rotor common to the two motors. However, the genuine rotary directions of the two motors are opposite to each other. Thus, when the proton motive force (PMF) is greater than the free energy drop in ATP hydrolysis, F_o_ wins and lets F_1_ rotate in its reverse direction. The reverse rotation leads to the reversal of the ATP hydrolysis reaction in F_1_, and ATP is synthesized. Conversely, when the PMF is lower, F_1_ wins, and protons are pumped back by reverse rotation of F_o_. ATP-driven rotation has been characterized in detail, particularly for isolated F_1_ [5–9].

F_1_ alone, without F_o_, can synthesize ATP when its rotor is forced to rotate in the reverse direction by an artificially applied force. F_1_ is thus a reversible molecular machine that can interconvert chemical and mechanical energies in either direction. This has so far been shown for a subcomplex, α_3_β_3_γ, of F_1_ derived from a thermophile, *Bacillus* PS3 [10,11]. The whole ATP synthase of the thermophile (TF_o_F_1_), however, has performed rather poorly in the past in *in vitro* studies. The maximal turnover rate, *V*_max_, has been reported to be 0.1 s^−1^ at 36 °C [12], 1–3 s^−1^ at 40 °C [13–15] and up to 7 s^−1^ at 40 °C in the presence of cholesterol [16]. In line with the rather low activities, reported Michaelis–Menten constants, *K*_m_, for substrates are high: 0.3 mm for ADP and 10 mm for P_i_ [14], or 0.4 mm for ADP and 6.3 mm for P_i_ [15]. ATP synthases from other sources generally show an activity more than an order of magnitude higher, and *K*_m_ values are correspondingly lower [17–21].

Because the thermophilic enzyme is robust and suited to single-molecule studies [3,5–8,10,11], we investigated whether TF_o_F_1_ with high synthesis activity can be prepared. The ε-subunit, in particular its C-terminal domain, exerts an inhibitory effect both for ATP hydrolysis and ATP synthesis, and deletion of this domain has been shown to increase the synthesis activity [22,23], probably by preventing the formation of the inhibited form. We thus sought for a reconstitution method that leads to a high synthesis activity. We obtained an activity of ∼ 16 s^−1^ at 30 °C, with a temperature coefficient that suggests a much higher activity at the physiological temperature of the thermophile. *K*_m_ values for the substrates at 30 °C were low and comparable with those of other enzymes, such that, unless *K*_m_ values at physiological temperatures differ significantly, efficient ATP synthesis will be ensured *in vivo*. In addition, the activity at room temperature (25 °C) of ∼ 10 s^−1^ suggests, on the basis of three ATPs per revolution [24], a rotary rate of ∼ 3 revolutions s^−1^, which should be readily detected in single-molecule studies under a microscope.

## Results

### ATP synthesis by mutant TF_o_F_1_ lacking the C-terminal domain of the ε-subunit (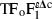) reconstituted into liposomes

A problem in the previous assays was the inhibitory effect of the ε-subunit on the ATP synthesis activity. In the absence of a nucleotide in the medium, TF_o_F_1_ is resting in a state inhibited by the ε-subunit [25], and recent studies suggest the possibility that activation of such TF_o_F_1_ to initiate ATP synthesis requires an extra PMF in addition to the thermodynamically required magnitude of PMF [26,27]. 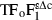, in which the C-terminal region of the ε-subunit that is responsible for the inhibitory effect is deleted, has shown a higher rate of ATP synthesis [22], and this was also the case for *Escherichia coli* F_o_F_1_ [23]. In this work, therefore, we prepared 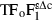, using as the wild type TF_o_F_1_ with a 10-histidine tag at each β-subunit (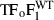) [28] (see Experimental procedures). Unless stated otherwise, all results below refer to 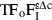.

We also improved the assay system to obtain high ATP synthesis activities reproducibly. Previously, TF_o_F_1_ was dissolved in solutions containing Triton X-100 during purification and proteoliposome reconstitution procedures [13–15]. However, we found that TF_o_F_1_ exposed to Triton X-100 has a strong propensity to form aggregates. In the improved assay, the TF_o_F_1_ preparation was dispersed in 6% n-octyl-β-d-glucoside (OG) in the presence of phospholipids, and OG was then removed with Biobeads (see Experimental procedures). The proteoliposomes thus made were very stable, and they retained 90% of ATP synthesis activity after storage for 3 days at 4 °C. This method is simple, does not require preformed liposomes, and is highly reproducible.

The ATP synthesis activity of the proteoliposomes was assayed by acid–base transition. First, the proteoliposomes were equilibrated with an acidic buffer with low K^+^ to set the pH inside the liposomes (pH_in_) to 5.65 and the internal K^+^ concentration ([K^+^]_in_) to 0.6 mm. The acidic buffer contained valinomycin to render the membranes permeable to K^+^. Then, the proteoliposomes were injected into a basic mixture to change the pH outside the liposomes (pH_out_) to 8.8 and the external K^+^ concentration ([K^+^]_out_) to 105 mm. This would generate a transient PMF of 330 mV, with the calculated membrane potential (Δψ) of 135 mV ([K^+^]_out_ = 105 mm, [K^+^]_in_ = 0.6 mm) and ΔpH of 3.2 (pH_out_ = 8.8, pH_in_ = 5.65). For detection of ATP, the reaction mixture contained luciferin and luciferase.

**Fig. 1:**
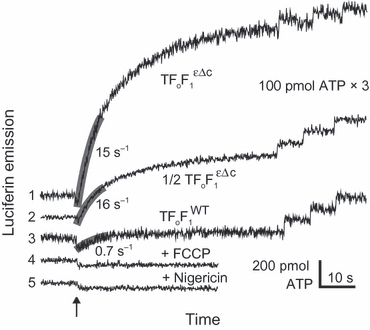
ATP synthesis by 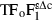 or 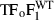 reconstituted in liposomes. The ATP synthesis reaction was initiated by injection of 100 μL of the acidified proteoliposome mixture into 900 μL of the basic mixture at the point indicated by the arrow (time zero), and luciferin emission was monitored at 30 °C. The final concentrations of TF_o_F_1_, ADP and P_i_ were 17 nm (8.5 nm in trace 2), 0.5 and 10 mm, respectively. At 60 s, 100 pmol of ATP was added three times for calibration. The imposed PMF calculated from the Nernst equation is 330 mV (pH_out_ = 8.8, pH_in_ = 5.65, [K^+^]_out_ = 105 mm, [K^+^]_in_ = 0.6 mm). The rate of ATP synthesis at time zero was estimated from the exponential fit for 0 – 6-s (thick gray curves on the experimental traces). Trace 1: 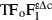. Trace 2: 1/2 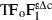. Trace 3: 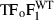. Trace 4: 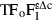 + FCCP. Trace 5: 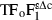 + nigericin. Other experimental details are described in Experimental procedures.

Figure 1 shows the time courses of the luciferase-catalyzed light emission, which directly reflected the increase in the ATP concentration resulting from synthesis by 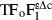. At the time indicated by the arrow (time zero), the proteoliposome mixture was injected into the basic mixture. ATP synthesis started at the maximum initial rate, which gradually slowed down and leveled off at ∼ 60 s, reflecting dissipation of the imposed PMF (time constant of the order of 10 s at 30 °C). To determine the activity at the calculated PMF of 330 mV, we estimated the initial velocity of ATP synthesis at time zero by fitting the 0–6-s portion with a single exponential (gray curves in Fig. 1) and converting the velocity to the turnover rate. As can be seen, the initial velocity of synthesis was proportional to the amount of the added proteoliposomes (Fig. 1, traces 1 and 2), giving similar rates of ATP synthesis by 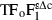 of 15 s^−1^ (trace 1) and 16 s^−1^ (trace 2). Under the same conditions, 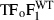 showed only low activity of 0.7 s^−1^ (trace 3). The low activity is consistent with previous studies with 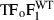, including one that used acid–base transition to obtain a rate of ∼ 2 s^−1^ at 40 °C [13]. No ATP synthesis was observed when an uncoupler, carbonyl cyanide 4-(trifluoromethoxy)phenylhydrazone (FCCP), was included in the mixture. Nigericin, which acts as an uncoupler in the presence of valinomycin, also abolished ATP synthesis.

Note that the orientation of the enzyme in the reconstituted membrane was not controlled for in this work. We did not apply correction for misoriented TF_o_F_1_, and thus the activity values reported here are probably underestimated. Also note that the catalyzing F_1_ was always exposed to the fixed pH_out_ of 8.8, and the activity values refer to the catalysis at this pH.

### Dependence on protein/lipid ratio

**Fig. 2:**
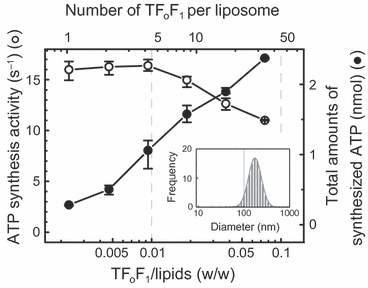
Effect of 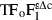/lipid ratio on ATP synthesis activity. Proteoliposomes were made by mixing 20–600 μg of 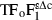 and 8 mg of lipid in 500 μL and adding Biobeads. The initial rate of synthesis and the amount of ATP synthesized by 50 s are shown. The imposed PMF was 330 mV (pH_out_ = 8.8, pH_in_ = 5.65, [K^+^]_out_ = 105 mm, [K^+^]_in_ = 0.6 mm). The scale at the top is based on the average proteoliposome diameter of 170 nm. The inset shows the size distribution estimated by dynamic light scattering.

To explore optimal conditions for activity assays, we prepared proteoliposomes with a fixed amount of phospholipid (16 mg·mL^−1^) and varying amounts of 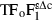, and measured the ATP synthesis activity (Fig. 2). The activity was almost constant, ∼ 16 s^−1^, for the 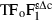/phospholipid weight ratio of 0.002 to 0.01. These ratios correspond to one to three molecules of 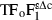 per proteoliposome of diameter 170 nm (Fig. 2, inset), a size expected for liposomes prepared in similar ways [29,30]. Beyond this range, the activity started to decrease gradually, although the total amount of ATP synthesized by 50 s increased steadily, at least to the weight ratio of 0.08. Because the measurement accuracy critically depends on the absolute amount of ATP synthesized, in the following experiments we used the proteoliposomes with a weight ratio of 0.02 (final 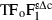 concentration in the reaction mixture of 17 nm).

### Dependence on temperature

**Fig. 3:**
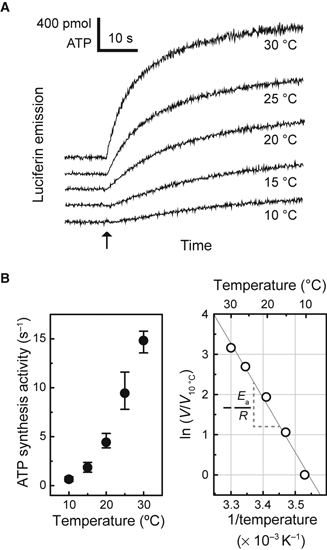
Temperature dependence of ATP synthesis activity. Activity was measured at 10, 15, 20, 25 and 30 °C (± 0.5 °C) under a PMF of 330 mV (pH_out_ = 8.8, pH_in_ = 5.65, [K^+^]_out_ = 105 mm, [K^+^]_in_ = 0.6 mm). (A) Time courses of ATP synthesis. (B) The initial (or maximal) ATP synthesis activity as a function of temperature (left), and the corresponding Arrhenius plot (right). *V*, activity; *R*, gas constant; *E*_a_, activation energy, which was 110 kJ·mol^−1^ in the range examined.

The results in Figs 1 and 2 were obtained at 30 °C. To determine the activity at the physiological growth temperature of *Bacillus* PS3 (∼ 60 °C or above) and to investigate the possibility of single-molecule experiments at room temperature, we examined the temperature dependence of the ATP synthesis activity. Unfortunately, the luciferase system was not perfectly stable above 30 °C, so we analyzed the activity between 10 and 30 °C (Fig. 3). Lowering the temperature greatly decreased the initial rate of synthesis, but the rate after 60 s did not differ much (Fig. 3). At 10 °C, synthesis of ATP started after a short lag. The reaction of luciferin/luciferase was sufficiently fast (∼ 0.1 s) at 10 °C, and the reason for the lag is unknown. There may also be a slight lag at 15 °C. We ignored these lag phases, and estimated the maximal rates of ATP synthesis (Fig. 3B). The activity increased three-fold to four-fold per 10 °C, or the *Q*_10_ temperature coefficient was 3–4 in this range. The Arrhenius plot (Fig. 3B, right) indicates an activation energy of 110 kJ·mol^−1^ in this range, and simple extrapolation would suggest an activity at the physiological temperature (∼ 60 °C) of ∼ 1000 s^−1^. Although such an extrapolation is not warranted, the physiological activity is probably above 100 s^−1^.

### Dependence on substrate concentrations

**Fig. 4:**
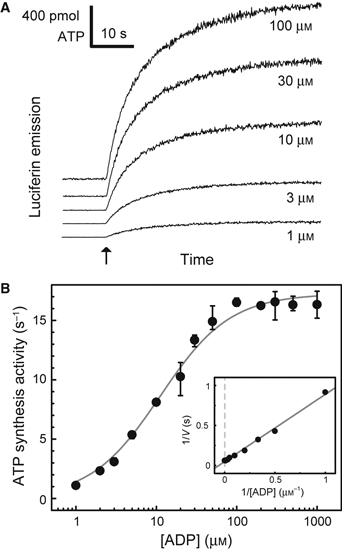
ADP dependence of synthesis activity. Activity was measured at 30 °C in the presence of a saturating P_i_ concentration of 10 mm under an imposed PMF of 330 mV (pH_out_ = 8.8, pH_in_ = 5.65, [K^+^]_out_ = 105 mm, [K^+^]_in_ = 0.6 mm). (A) Time courses. (B) The initial activity versus ADP concentration. The line shows a Michaelis–Menten fit with 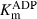 = 13 μm and *V*_max_ = 17 s^−1^. Inset: Lineweaver–Burk plot.

**Fig. 5:**
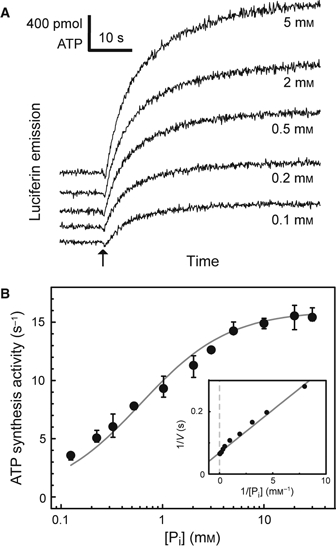
Phosphate dependence of synthesis activity. Activity was measured at 30 °C in the presence of a saturating ADP concentration of 0.5 mm under an imposed PMF of 330 mV (pH_out_ = 8.8, pH_in_ = 5.65, [K^+^]_out_ = 105 mm, [K^+^]_in_ = 0.6 mm). The amount of contaminant P_i_ was 25 μm, and is not corrected for. (A) Time courses. (B) Phosphate dependence. The line shows a Michaelis–Menten fit with 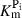 = 0.55 mm and *V*_max_ = 16 s^−1^. Inset: Lineweaver–Burk plot.

At 30 °C, we examined how substrate concentrations affect the rate of ATP synthesis. The ADP concentration was changed from 1 μm to 1 mm at a saturating concentration (10 mm) of P_i_ (Fig. 4). The data are fitted well with the Michaelis–Menten equation with a 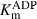 of 13 μm and a *V*_max_ of 17 s^−1^. We also changed the P_i_ concentration from 0.1 to 30 mm at a saturating concentration (0.5 mm) of ADP. The results also conformed to the Michaelis–Menten equation, with a 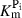 of 0.55 mm and a *V*_max_ of 16 s^−1^ (Fig. 5).

## Discussion

We have developed simple and reproducible procedures for the preparation of active TF_o_F_1_ proteoliposomes and conditions for real-time monitoring of ATP synthesis. The synthesis activity reported here is an order of magnitude higher than that in previous reports on TF_o_F_1_ [12–16]. Note that most of the previous work was performed at 40 °C, whereas our measurements here were made at 30 °C. The primary reason for the increase in activity is the removal of the inhibitory C-terminal segment of the ε-subunit, as seen in Fig. 1. In addition, we noticed that complete solubilization of TF_o_F_1_ with proper detergents and a low protein/lipid ratio are keys to high activity. Also, Biobeads need to be selected from among several lots to obtain maximal activity under the protocol described here, or else the amount of added Biobeads and incubation time should be optimized for each lot.

The Michaelis–Menten constants for the substrates, 13 μm for ADP and 0.55 mm for P_i_, obtained here are low enough to ensure efficient ATP synthesis under cellular conditions where the ADP concentration is expected to be submillimolar and the P_i_ concentration several millimolar. There is no guarantee that the *K*_m_ values at the physiological temperature of the thermophile are close to our experimental values at 30 °C, but the lower *K*_m_ values are more advantageous than the previous values of 0.3–0.4 mm for ADP and 6–10 mm for P_i_ [14,15]. These previous values may, in part, reflect the properties of the ε-subunit-inhibited fraction. It is also possible that ADP and/or P_i_ help to convert the inhibited form to an active form, and the measured *K*_m_ might be influenced by these activation processes.

As noted above, the reported ATP synthesis activity of TF_o_F_1_ has so far been much lower and the *K*_m_ values for ADP and P_i_ higher than those of F_o_F_1_ from other sources. Bovine enzyme in submitochondrial particles gave, in its high-activity mode, a *V*_max_ of ∼ 420 s^−1^ at 30 °C [17], a 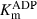 of 50–100 μm, and a 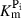 of ∼ 2 mm (PMF unknown) [18]. Yeast mitochondrial ATP synthase reconstituted in liposomes showed a *V*_max_ of 120 s^−1^ at 25 °C and an apparent 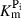 lower than 1.5 mm at a pH on the F_1_ side below 8 (PMF of 250–300 mV) [19]. The reconstituted chloroplast enzyme gave a *V*_max_ up to ∼ 400 s^−1^ and a 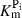 of 0.35 or 0.97 mm, depending on the reconstitution protocol (PMF of ∼ 300 mV) [20]. *E. coli* ATP synthase in liposomes showed a *V*_max_ of ∼ 30 s^−1^ at room temperature, a 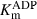 of 27 μm, and a 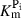 of 0.7 mm (PMF of ∼ 330 mV) [21]. Another report on the *E. coli* enzyme [23] gave a *V*_max_ of 16–20 s^−1^ at 24–25 °C, a 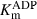 of 100 μm and a 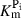 of 4 mm for the wild type, and a *V*_max_ of ∼ 60 s^−1^, a 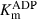 of 25 μm and a 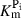 of 3 mm for an εΔC mutant (PMF of ∼ 260 mV). This last result obtained with the bacterial enzyme is qualitatively similar to that obtained with TF_o_F_1_, in that C-terminal truncation of the ε-subunit increases *V*_max_ while decreasing *K*_m_ values for ADP and P_i_. The present results on TF_o_F_1_ place this thermophilic enzyme among those with regular synthesis activities, and, with regard to *K*_m_ values, at the low end. Note that the *V*_max_ of TF_o_F_1_ at its physiological temperature of ∼ 60 °C or above is expected to be much higher than 16 s^−1^ (Fig. 3).

The demonstration of substantial ATP synthesis by TF_o_F_1_ around room temperature should be a large step towards single-molecule observation of rotation-catalyzed ATP synthesis under an optical microscope. The thermophilic enzyme is quite stable, remaining active for days at room temperature. This stability greatly facilitates microscopic work, which is tedious both in preparation and observation (both take hours). Indeed, much of the mechanical characterization of F_1_ has been achieved with F_1_ derived from the thermophile, *Bacillus* PS3. We hope to answer, by using TF_o_F_1_, the fundamental questions of how protons rotate F_o_F_1_ and how rotation leads to ATP synthesis. So far, even the demonstration of proton-driven rotation has been difficult [31], but a major obstacle, the low activity, has now been removed.

## Experimental procedures

### Preparation of TF_o_F_1_

In this work, we used TF_o_F_1_ with a 10-histidine tag at the N-terminus of each β-subunit [25] as the wild type (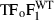). The mutant lacking the C-terminal domain of the ε-subunit (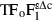) was produced by inserting a stop codon after ε-Asp87. 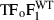 and 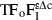 were expressed in an F_o_F_1_-deficient *E. coli* strain (DK8) with the expression plasmids pTR19-ASDS and pTR19-ASDS-εΔc, respectively, and purified as previously described [25], with the following modifications. The membrane fraction containing TF_o_F_1_ was solubilized at 30 °C in a solution containing 10 mm Hepes, 5 mm MgCl_2_, 10% (v/v) glycerol, 0.5% (w/v) cholic acid and 2% (v/v) Triton X-100, with the pH adjusted to 7.5 with KOH. The suspension was centrifuged at 235 000 ***g*** for 60 min. The supernatant was diluted six-fold with M-buffer (20 mm KP_i_ and 100 mm KCl, pH 7.5). To this solution, Ni^2+^–Sepharose resin (GE Healthcare, Uppsala, Sweden) that had been pre-equilibrated with W-buffer [M-buffer containing 20 mm imidazole and 0.15% (w/v) n-decyl-β-d-maltoside (Dojindo, Kumamoto, Japan), with the pH adjusted to 7.5 with HCl] was added, and the suspension was gently stirred on ice for 30 min. The resin suspension was then poured into an open column and washed with 10 volumes of W-buffer. Protein was eluted with M-buffer containing 200 mm imidazole and 0.15% n-decyl-β-d-maltoside, with the pH adjusted to 7.5 with HCl, and diluted three-fold with 20 mm Hepes, 0.2 mm EDTA and 0.15% n-decyl-β-d-maltoside, with the pH adjusted to 7.5 with NaOH. The suspension was applied to a RosourceQ column (6 mL; GE Healthcare) equilibrated with the same buffer. Elution with a linear gradient of 0–500 mm Na_2_SO_4_ produced two closely located protein peaks. The second peak contained TF_o_F_1_, which was concentrated by a centrifugal concentrator with a cut-off molecular mass of 50 kDa (Amicon Ultra; Millipore, Country Cork, Ireland) to a final volume of ∼ 1 mL. The purified TF_o_F_1_ preparation was divided into aliquots of 25–50 μL, frozen with liquid N_2_, and stored at −80 °C until use. The molar concentration of TF_o_F_1_ was determined from absorbance with a molar extinction coefficient at 280 nm of 253 000 m^−1^ cm^−1^. Protein mass was calculated by taking the molecular mass of TF_o_F_1_ as 530 kDa.

### Reconstitution of TF_o_F_1_ into liposomes

Crude soybean l-α-phosphatidylcholine (Type II-S; Sigma, St. Louis, MO, USA) was washed with acetone [32] and suspended to a final concentration of 32 mg·mL^−1^ in R-buffer (20 mm Tricine, 20 mm succinic acid, 80 mm NaCl and 0.6 mm KOH, with the pH adjusted to 8.0 with NaOH). The suspension was incubated for 30 min with gentle stirring, to allow the lipid to swell. The lipid was further dispersed by brief sonication with a tip-type sonicator (UR-20P; Tomy Seiko, Tokyo, Japan) for 30 s. This suspension was divided into aliquots, frozen with liquid N_2_, and stored at – 80 °C until use. Reconstitution of TF_o_F_1_ into liposomes was performed as follows. The lipid suspension (250 μL) was mixed with 250 μL of TF_o_F_1_ in R-buffer containing 10 mm MgCl_2_ and 12% (w/v) OG, and the mixture (total volume, 500 μL; concentration of TF_o_F_1_, 40–1200 μg·mL^−1^) was stirred gently at 25 °C for 1 h. To this solution, 200 μL of Biobeads (SM-2; BioRad, Hercules, CA, USA), which had been pre-equilibrated with R-buffer, was added. The mixture was stirred gently for 30 min at 25 °C, and 300 μL of Biobeads was added to the mixture. After another 1.5 h of incubation, the liposome suspension was transferred to a new tube, leaving the Biobeads behind. The concentration of TF_o_F_1_ in the final mixture was 75–2300 nm. The average diameter of the proteoliposomes was estimated by dynamic light scattering (HB-550; Horiba, Kyoto, Japan) to be 170 nm (Fig. 2).

### ATP synthesis assay and data analysis

ATP synthesis by TF_o_F_1_ was monitored with a luciferase assay, as previously described [33], in a luminometer (Luminescencer AB2200; ATTO, Tokyo, Japan) equipped with a sample injection apparatus. The synthesis reaction was driven by acid–base transition and valinomycin-mediated K^+^-diffusion potential as follows. A basic mixture was prepared by mixing 21 μL of the luciferin/luciferase mixture (2 × concentration, ATP bioluminescence assay kit CLSII; Roche, Mannheim, Germany), 870 μL of B-buffer (200 mm Tricine, 10 mm NaH_2_PO_4_, 2.5 mm MgCl_2_ and 120 mm KOH, with the pH adjusted to 8.8 with NaOH) and 9 μL of 50 mm ADP (A-2754; Sigma), and was incubated for 5 min at 30 °C. In experiments for the determination of *K*_m_, the concentration of NaH_2_PO_4_ above was varied between 0.1 and 30 mm (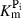), and the concentration of ADP between 1 μm and 1 mm (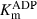). In a separate tube, the proteoliposome suspension (30 μL) was mixed with 68 μL of an acidic buffer (A-buffer: 20 mm succinic acid, 14.7 mm NaH_2_PO_4_, 2.5 mm MgCl_2_ and 0.6 mm KOH, with the pH adjusted to 5.1 with NaOH), 1 μL of 50 mm ADP and 1 μL of 20 μm valinomycin in ethanol. In assays for *K*_m_, the NaH_2_PO_4_ concentration above was varied between 0.147 and 44.1 mm, and the ADP concentration between 1 μm and 1 mm. The resultant proteoliposome mixture was incubated for 5 min at 30 °C to allow equilibration across the membrane. Inclusion of ADP in the proteoliposome mixture improved ATP synthesis activity about two-fold. The ATP synthesis reaction was initiated by injecting 100 μL of the proteoliposome mixture into 900 μL of the basic mixture in the luminometer with a syringe (LC-100; Kusano, Tokyo, Japan), and the change in luciferin emission was monitored continuously. When indicated, 200 nm FCCP or 500 nm nigericin in ethanol was included in the reaction mixture. At the end of the reaction (60 s), 10 μL of 10 μm ATP was added three times for calibration. The ADP solution that we used contained ATP amounting to 0.05% or 0.2% ADP, depending on the lot, as determined by the luciferase assay. The amount of contaminating P_i_ in the reaction mixture was 25 μm as assessed with the EnzChek Phosphate Assay Kit (Invitrogen, Eugene, OR, USA). Unless otherwise indicated, the final concentrations of TF_o_F_1_, ADP and P_i_ in the reaction mixture were 17 nm, 0.5 mm and 10 mm, respectively. The activity values reported are the average over three to five measurements, each with a different preparation in most cases, and the error bars in the figures show the range. The pH values of the reaction mixture and the acidified proteoliposome mixture, termed pH_out_ and pH_in_, respectively, were measured with a glass electrode, and ΔpH is defined as (pH_out_ − pH_in_). The membrane potential was calculated from the Nernst equation, Δψ = (*RT*/*F*)ln([K^+^]_out_/[K^+^]_in_) or 60 · log([K^+^]_out_/[K^+^]_in_) in millivolts for our experiments at 30 °C. The magnitude of the PMF is given (in mV) as 60 · ΔpH + Δψ.

### Calculation of *K*_m_

*K*_m_ values were estimated by nonlinear fit with origin (OriginLab). The synthesis activity, *V*, was fitted with the equation *V* = (*V*_max_· [S])/(*K*_m_ + [S]), where S is ADP or P_i_.

## Abbreviations

Δψ: membrane potential
FCCP: carbonyl cyanide 4-(trifluoromethoxy)phenylhydrazone
F_o_F_1_: F_o_F_1_-ATPase
[K^+^]_in_: internal K^+^ concentration
[K^+^]_out_: external K^+^ concentration
OG: n-octyl-β-d-glucoside
pH_in_: pH inside the liposomes
pH_out_: pH outside the liposomes
PMF: proton motive force
TF_o_F_1_: *Bacillus* PS3 F_o_F_1_-ATPase
mutant *Bacillus* PS3 F_o_F_1_-ATPase lacking the C-terminal domain of the ε-subunit
*Bacillus* PS3 wild-type F_o_F_1_-ATP synthase
